# Administration of Adenosine Triphosphate Provides Additional Value Over Programmed Electrophysiologic Study in Confirmation of Successful Ablation of Atrioventricular Accessory Pathways

**DOI:** 10.3389/fcvm.2021.716400

**Published:** 2021-11-16

**Authors:** Wei Wei, Xianhong Fang, Michael Shehata, Xunzhang Wang, Xianzhang Zhan, Hai Deng, Hongtao Liao, Zili Liao, Yang Liu, Yumei Xue, Shulin Wu

**Affiliations:** ^1^Guangdong Cardiovascular Institute, Guangdong Provincial People's Hospital, Guangdong Academy of Medical Sciences, Guangzhou, China; ^2^Guangdong Provincial Key Laboratory of Clinical Pharmacology, Guangdong Provincial People's Hospital, Guangdong Academy of Medical Sciences, Guangzhou, China; ^3^Cedars Sinai Medical Center, Heart Institute, Los Angeles, CA, United States

**Keywords:** adenosine triphosphate, programmed electrophysiologic study, catheter ablation, accessory pathways, long-term outcomes

## Abstract

**Objectives:** To study the benefit of adenosine triphosphate (ATP) in evaluating ablation endpoints of accessory pathways (AP) and subsequent long-term prognosis.

**Methods:** We reviewed consecutive patients with supraventricular tachycardias due to APs that underwent radiofrequency catheter ablation (RFCA) from January 2016 to September 2018 in our center. The patients were divided into two groups: the ATP group (who had passed both the ATP test and PES after ablation as the endpoint) and the non-ATP group (who had passed PES only after ablation as the endpoint). We reviewed the patients' intra-cardiac electrograms and analyzed their long-term outcomes.

**Results:** In total, 1,343 patients underwent successful RFCA. There were 215 patients in the ATP group with one lost to follow-up. There were 1,128 patients in the non-ATP group with 39 lost to follow-up. Twenty-three patients in the ATP group demonstrated additional electrophysiological entities due to ATP administration, including reappearance of the ablated APs in 16 patients, discovery of PES-undetected APs in 5, induction of atrial fibrillation in 5, premature atrial contractions in 1, and premature ventricular contractions in another. During the 7 to 39 months (average 24.4 ± 9.5 months) follow-up, the recurrence rate was 8.41% (18/214) in the ATP group and 6.80% (74/1,084) in the non-ATP group. In subjects with recurrence, 14 patients (14/18 = 77.8%) in the ATP group and 50 patients (50/74 = 67.6%) in the non-ATP group accepted redo ablations. Among the ATP-group, all the 14 redo APs were the old ones as before. Among the non-ATP-group, redo ablations confirmed that 39 APs were the old ones, while 20 APs were newly detected ones which had been missed previously. The difference in recurrent AP locations confirmed by redo procedures between the two groups was significant (*p* = 0.008). In the non-ATP group, 20 (40%) of redo cases were proven to have multiple APs, while 33 (3.3%) cases who did not suffer from recurrence had multiple APs. Existences of multiple APs in recurred cases were significantly higher than that in non-recurred ones in the non-ATP group (*p* < 0.001), while there was no such difference in the ATP group (*p* = *0.114*).

**Conclusions:** The existence of multiple APs was more common in recurrent cases if ATP was not used for confirmation of ablation endpoints. ATP probably has additional value over PES alone by detecting weak AP conductions. ATP can evoke atrial and ventricular arrhythmias.

## Introduction

There are usually two methods to determine successful ablation endpoints for accessory pathways (AP). One is programmed electrophysiological study (PES), the other is medication confirmation, including injection of isoproterenol which can activate sympathetic nerves and adenosine/adenosine triphosphate (ATP) which can block the atrioventricular (AV) node to feature the existence of APs if still there (1, 2). Catecholamine sensitivity is an uncommon feature of APs (3). Although isoproterenol can change reentrant coupling between APs and the AV node, it seldom affects APs directly, except for a few isoproterenol-sensitive APs (4). Adenosine and ATP have been used for determination of AP ablation endpoints for years, but previous studies were mostly cross-sectional and seldom provided follow-up data (5, 6). Many electrophysiologists now prefer to do PES alone to test the endpoint since PES is considered adequate to determine the presence of most APs especially when they could be easily detected before ablation. We evaluated that the administration of ATP in combination with PES could be superior to PES alone in confirmation of ablation endpoints of APs, based on long-term follow-up data.

## Methods

### Patient Population and Pre-procedural Preparation

We reviewed consecutive cases with supraventricular tachycardias due to APs demonstrated by intracardiac PES that underwent radiofrequency ablation from January 2016 to September 2018 in our hospital. All patients were older than 14 years of age and all of them or their guardians had signed informed consents for invasive PES and ablation before the procedure. We divided the patients into two groups: those who had passed both PES and ATP testing for the absence of an AP as the endpoint of catheter ablation (ATP group) and those without administration of ATP (non-ATP group) that underwent PES alone. Contraindications to ATP such as asthma, bronchitis, chronic obstructive pulmonary diseases, heart failure, uncontrolled hypertension, or significant ischemia were excluded before the ATP test. The following patients were excluded from the analysis: 1. Those who had experienced previous AP ablations before January 1, 2016, 2. patients with acute unsuccessful outcomes of AP ablations in our hospital, 3. patients who did not consent to AP ablation following PES.

### Electrophysiological Study

Two quadripolar mapping catheters including a His bundle catheter, and right ventricular apex (RVA) catheter were placed via the right femoral vein under fluoroscopic guidance. A decapolar (2-8-2 mm inter-electrode space) coronary sinus (CS) vein catheter was placed either via the left/right femoral vein or via the left sub-clavicular vein. Intracardiac electrocardiograms during sinus rhythm and arrhythmia were analyzed. Electrophysiological study techniques including burst pacing, programmed stimulation, entrainment, and His bundle refractory premature ventricular stimulus (RS2) were used for diagnosis of the arrhythmia mechanism.

We divided the A-V annulus into 12 parts: I. Left posterior septum, II. left posterior free wall, III. left lateral free wall, IV. left anterior free wall, V. coronary sinus orifice, VI. right posterior septum, VII. right inferior free wall, VIII. right superior free wall, IX. epicardial posterior septum within the coronary sinus, X. His region, XI. aorto-mitral continuity, and XII. nodo-ventricular fibers. Divided AP locations are shown in Supplementary Figure 1.

### Ablation and Procedure Endpoints

Three-dimensional electro-anatomical mapping systems (Carto 3, Biosense Webster, Diamond Bar, CA and Ensite NAVX, Abbott, Minneapolis, MN) were used for most procedures, while some procedures were guided only by fluoroscopy. Temperature-controlled ablation catheters were applied to most patients (55–60°C, 40–70 W), while irrigated catheters were used in some challenging cases (43°C, 20–40 W, saline flow 17–30 mL/min). The ablation targets were located on the atrio-ventricular annulus with the earliest local atrial potential during ventricular pacing or tachycardia for concealed APs, and the earliest local ventricular potential for manifest APs, with AP potentials if available. For nodo-ventricular fibers, ablation was done at the slow AV nodal pathway area, the lower region of the Triangle of Koch.

For the non-ATP group, after successful ablation of the AP, PES was conducted including S1S1, S1S2, and S1S2S3 stimulation in the atrium and ventricle on the ipsilateral side and the contralateral site of the AP in turn by the ablation catheter or the RVA catheter. If no antegrade A-V conduction (pre-excitation) or retrograde V-A conduction via APs (V-A dissociation during ventricular pacing) was observed, the procedure was considered successful and concluded after at least a 30-min waiting time in total post-ablation.

For the ATP group, if the patients had passed the above PES after initial ablation without evidence of AP, subjects underwent administration of the ATP test. Ventricular pacing was used temporarily with either the RVA catheter or the ablation catheter to ensure the right/left ventricle could be captured as a protection against slow ventricular rhythm during the ATP test. An ATP bolus was rapidly injected via the peripheral vein to block the AV node. If the patient's body weight was <50 kg, a lower bolus of 20 mg of ATP was injected. If the patient's body weight was more than 75 kg, a higher bolus of 40 mg of ATP was injected. Otherwise, a bolus of 30 mg of ATP was injected. When block of AV node was observed without antegrade A to V conduction or reappearance of pre-excitation, ventricular pacing from the ipsilateral side of the AP was performed to detect retrograde V to A conduction as a sign of concealed AP conduction. The procedure was concluded if there was no evidence of an AP, confirmed by ATP testing plus PES.

### Follow-Up

All the patients were tele-connected to our follow-up staff by mobile application software which assisted in keeping in long-term touch with the medical aid of our center. Patients were advised to contact our follow-up staff if they had any recurrent symptoms and/or abnormal ECG findings. All the patients were routinely followed up over 1 month with a 12-lead surface ECG and inquiry of the existence of palpitations or other arrhythmia-related symptoms by EP faculty in the out-patient clinic. Afterwards, the follow-up staff would be consistently available to the patients *via* a mobile app. For any recurrent symptoms reported, patients would arrange visits to doctors in the out-patient clinic. Patients were also advised to go to the emergency room immediately whenever they suffered from recurrent symptoms to record real-time ECGs. Some patients accepted 24-h Holter monitoring. If confirmation of SVT was still obscure, they would endure trans-esophageal atrial pacing. Once recurrence was confirmed, the patient would be advised to have a redo ablation. Moreover, all the patients received telephone follow-ups between March 2019 and April 2019 for inquiry of long-term outcomes. Patients were considered free from recurrences if they fulfilled any one of the following criteria: 1. Negative ECG findings of delta waves without palpitations or dyspnea; 2. real-time ECG with symptoms but no evidence of supraventricular tachycardia and delta waves; and 3. negative findings of trans-esophageal pacing.

### Statistical Analysis

Measurement variables are presented as mean ± standard deviation. Categorical variables are presented as percentages. Redo procedures performed on the same patients were assumed to be independent. A Chi-squared test (Pearson's Chi-squared test or Fisher's exact test) was done for comparison of categorical variables between the ATP group and the non-ATP group and for comparison of factors related to recurrences within each group.

## Results

### General Data

**Figure 1:**
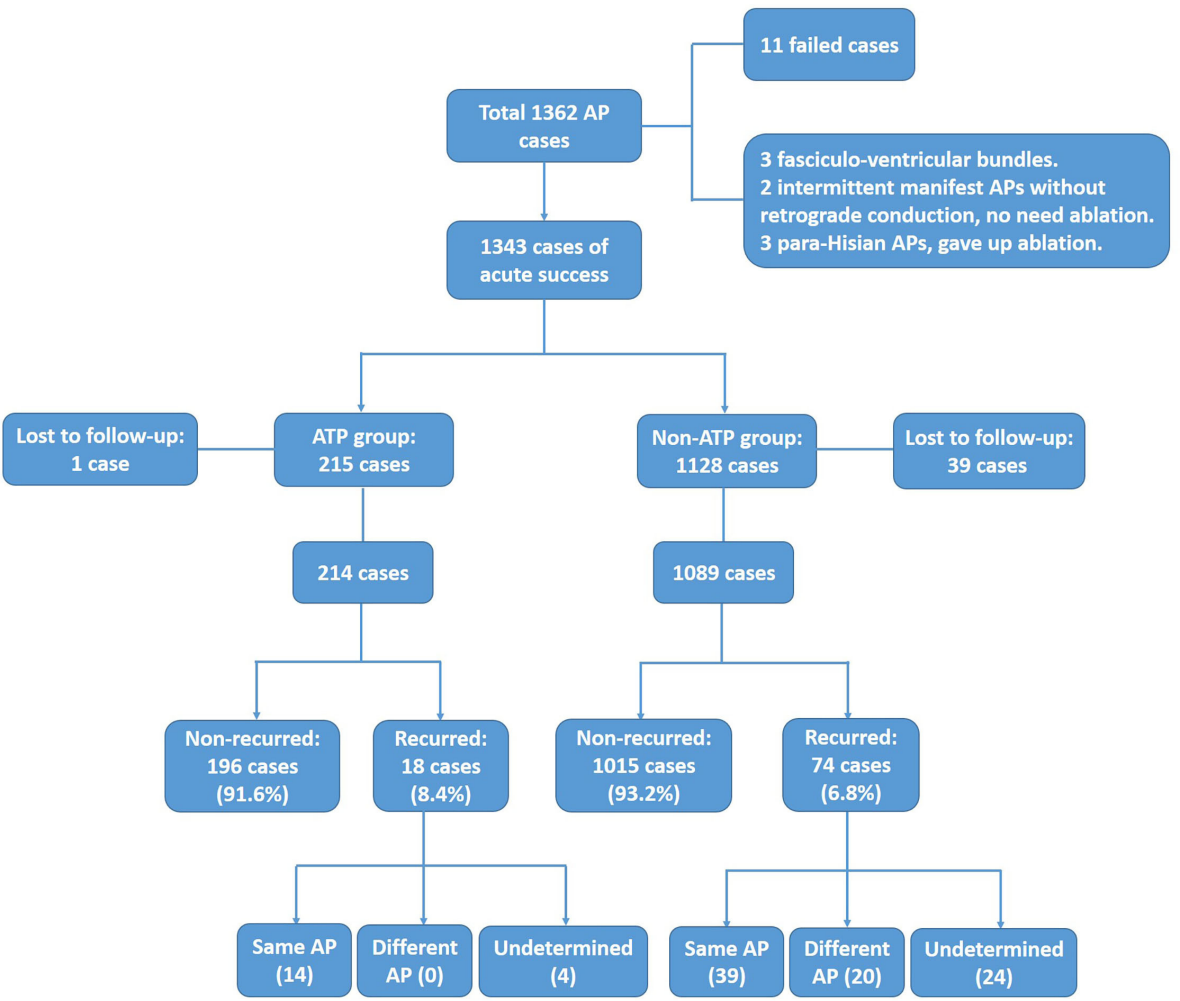
Enrollment of ATP group and non-ATP group. After excluding failed, unablated, and lost to follow-up cases, 214 and 1,089 cases were enrolled for final analysis in the ATP group and the non-ATP group, respectively.

There were a total of 1,362 patients with APs that underwent electrophysiology study during this period. Nineteen patients were excluded from further analysis, including 11 failed cases, 3 fasciculo-ventricular bundles that did not need ablation, 2 intermittent manifest APs without retrograde conduction that did not need ablation, and 3 para-Hisian APs where ablation was not performed. In the remaining 1,343 patients, 215 patients comprised the ATP group (1 lost to follow-up, 214 were enrolled in further statistics, mean age 36.8 ± 14.3 years) and 1,128 patients were within the non-ATP group (39 lost to follow-up, 1,089 enrolled in further statistics, mean age 41.4 ± 15.0 years). Three-dimensional electro-anatomical mapping systems (Carto 3 and Ensite NAVX) were used for 952 patients, while procedures for the other 391 patients were guided only by fluoroscopy. Temperature-controlled ablation catheters were applied in 1,304 cases (55–60°C, 40–70 W), while irrigated catheters were applied in 39 cases (43°C, 20–40 W, irrigation flow 17–30 mL/min). The enrollment flow chart is shown in Figure 1. Baseline data are shown in Table 1.

**Table 1:** General demographic data and clinical status of both groups.

**Factors**	**ATP group (*n* = 214)**	**Non-ATP group (*n* = 1,089)**	***P*-value**
Age, years	36.8 ± 14.3	41.4 ± 15.0	<0.001
Gender, %	M: 127 (58) W**:** 87 (42)	M: 649 (60) W: 440 (40)	0.946^a^
Body weight (kg)	60.6 ± 11.1	53.8 ± 11.5	<0.001
Body mass index (kg/m^2^)	22.2 ± 3.9	22.9 ± 5.0	0.182
Hypertension	17 (7.9)	91 (8.4)	0.841^b^
Diabetes	5 (2.3)	34 (3.1)	0.823^c^
Renal insufficiency	1 (0.5)	3 (0.3)	0.513^f^
Hyperglycemia or gout	7 (3.3)	29 (2.7)	0.620^d^
Baseline structural heart diseases Type of heart diseases	7 Coronary artery disease 1 Myocardial non-compaction 1 Mitral valve prolapse 1 Atrial septal defect 1 Ebstein's anomaly 1 Tricuspid insufficiency 1 Unclassified cardiomyopathy 1	57 Coronary artery disease 14 Mitral valve prolapse 9 Ebstein's anomaly 7 Hypertrophic cardiomyopathy 4 Rheumatic heart disease 4 Fallot tetralogy 1 Ventricular septal defect 1 Atrial septal defect 1 Aortic bicuspid valve 5 Left ventricle non-compaction 1 Tricuspid insufficiency 3 Aortic valve replacement 1 Restrictive cardiomyopathy 1 Myocarditis 2 Dilated cardiomyopathy 1 Unclassified cardiomyopathy 2	0.224^e^

*M, man; W, woman*.

a *Pearson Chi-Square value 0.005*.

b *Pearson Chi-Square value 0.04*.

c *Pearson Chi-Square value 0.265*.

d *Pearson Chi-Square value 0.246*.

e *Pearson Chi-Square value 1.476*.

f *Analyzed by Fisher's exact test*.

### Distributions of All the Detected APs in Both Groups During the First Procedure

**Figure 2:**
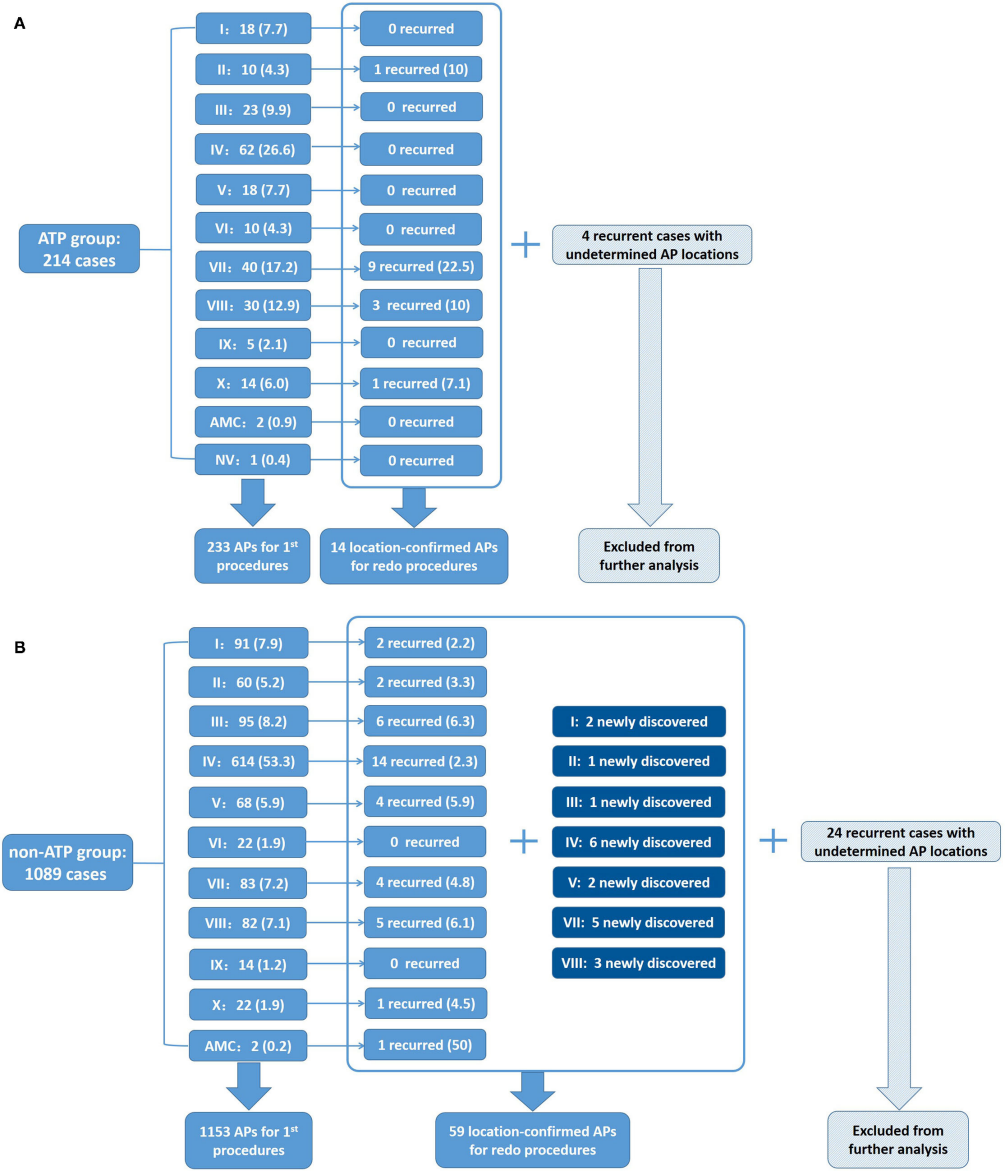
Distributions of APs detected in the ATP group and the non-ATP group. **(A)** Distribution percentages of APs in the ATP group in first procedures and recurrence percentages redo procedures of the ATP group. Four recurrent cases with undetermined AP locations were excluded from further analysis. **(B)** Distribution percentages of APs in the first procedures and recurrence percentages in redo procedures of the non-ATP group. Twenty-four recurrent cases with undetermined AP locations were excluded from further analysis.

The distributions of all the detected APs in both groups during the first procedure are shown in Figures 2A,B. AP distributions of the ATP group were: I-18 (7.7%), II-10 (4.3%), III-23 (9.9%), IV-62 (26.6%), V-18 (7.7%), VI-10 (4.3%), VII-40 (17.2%), VIII-30 (12.9%), IX-5 (2.1%), X-14 (6%), AMC-2 (0.9%), and NV-1 (0.4%), totaling 233 APs (100%). AP distributions of the non-ATP group were: I-91 (7.9%), II-60 (5.2%), III-95 (8.2%), IV-614 (53.3%), V-68 (5.9%), VI-22 (1.9%), VII-83 (7.2%), VIII-82 (7.1%), IX-14 (1.2%), X-22 (1.9%), and AMC-2 (0.2%), totaling 1,153 APs (100%) (Supplementary Table 1). The distributions of all the AP locations were significantly different between the two groups (Pearson Chi-square value = 81.785, *p* < 0.001).

### Phenomena Observed After ATP Administration

**Figure 3:**
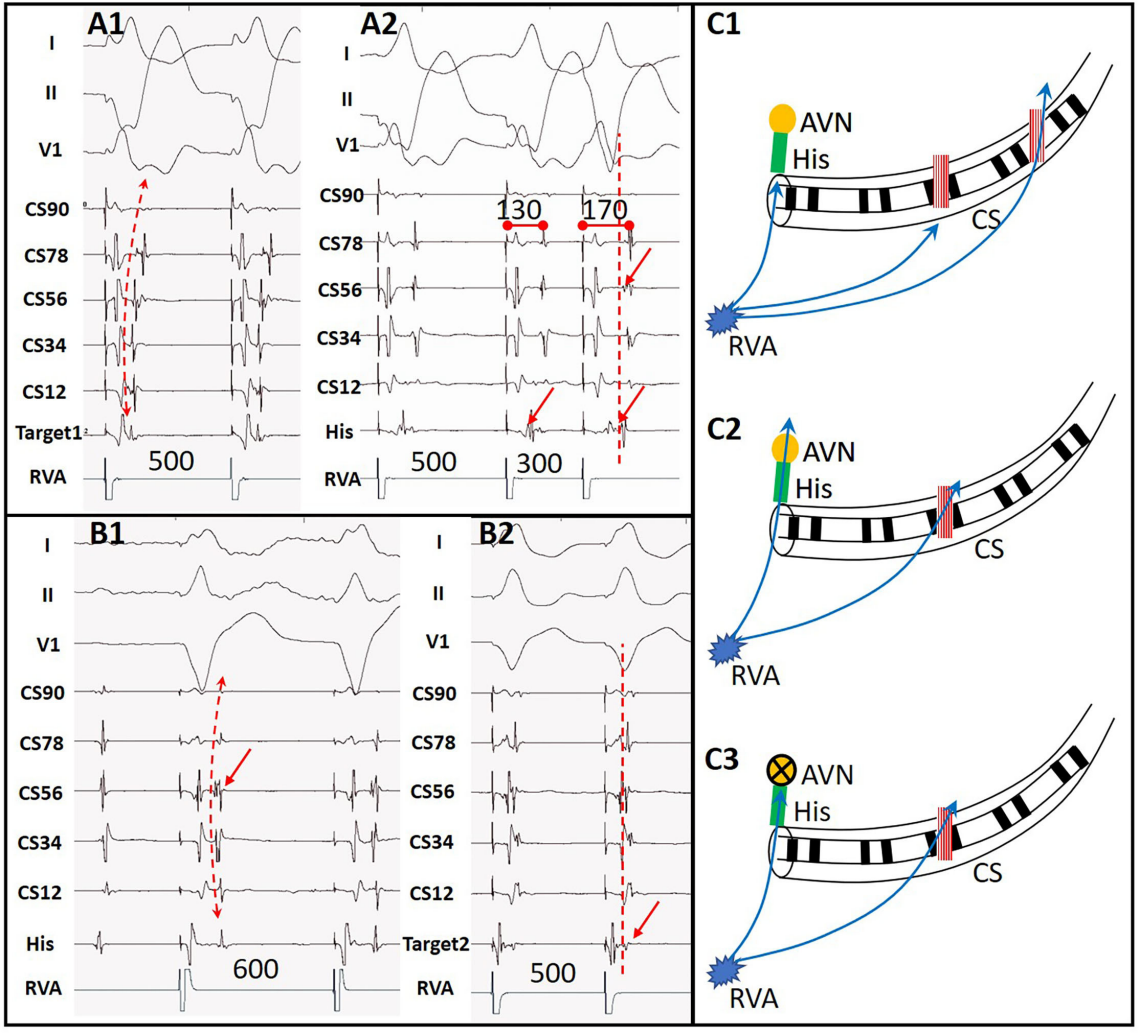
A case with another AP detected by ATP. **(A1)** Ventricular stimulation showed acentric retrograde V-A conduction indicating a left lateral wall AP. **(A2)** Decremental V-A conduction after ablation of the first AP with the earliest retrograde A at CS56. **(B1)** Retrograde V-A conduction during ATP functioning with the earliest retrograde A at CS56, indicating a second AP *in situ*. **(B2)** Target of the second AP near CS56. **(C1)** Sketch map of predominant retrograde conduction via the first AP at the left lateral wall during RVA pacing as **(A1)**. **(C2)** Simultaneous retrograde conduction via AVN and the second AP during RVA pacing after elimination of the first AP as **(A2)**. **(C3)** Retrograde conduction *via* the second AP at CS56 during ATP functioning as **(B1)**. RVA, right ventricular apex; AVN, atrio-ventricular node; CS, coronary sinus; His, His bundle.

**Figure 4:**
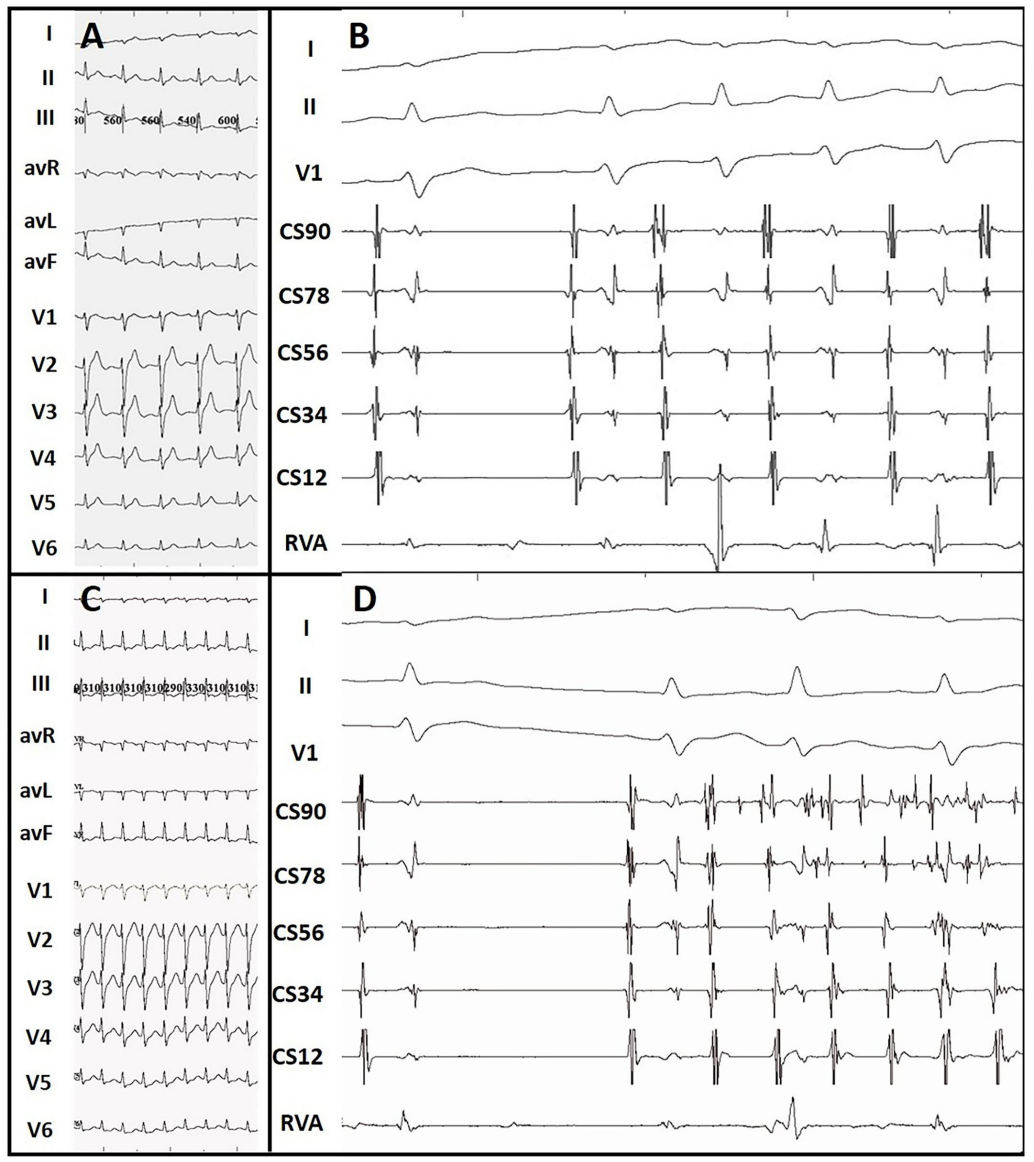
ATP-induced paroxysmal atrial tachycardia and atrial fibrillation. **(A)** Sinus surface ECG. **(B)** ATP-induced paroxysmal atrial tachycardia. **(C)** Surface ECG during supraventricular tachycardia. **(D)** ATP-induced paroxysmal atrial fibrillation. Atrial tachycardia and atrial fibrillation terminated within 10 s and 5 min, respectively.

Among the ATP group, 23 patients showed additional arrhythmia substrates after administration of ATP during their first procedures. Among them, 16 patients' ablated APs which had passed PES re-appeared after initial ablation. These APs were further ablated until passing both ablation endpoints. Five patients showed evidence of APs other than the ablated ones that had not been detected by PES (Figure 3). All these missing APs were further ablated and tested again. Five patients had paroxysmal atrial fibrillation (PAF, Figure 4) lasting from 15 s to 5 min. One patient had frequent premature atrial contractions (PAC) and PAC did not disappear when ATP faded away. PAC ablation was done at the same time after additional informed consent of the patient and her family was obtained since we considered it would likely happen repeatedly in the patient's daily life. One patient had frequent premature ventricular contractions (PVC) that subsequently abated within 5 min of ATP bolus. Details are shown in Supplementary Table 2. On retrospective review, there were two patients with AP re-appearance during ATP testing that failed to be identified before conclusion of the procedures and suffered from recurrence during follow-up. They accepted redo ablations after their previous intra-cardiac electrograms had been reviewed.

Almost all patients who underwent ATP testing experienced related symptoms, including chest pressure, dyspnea, or flushing. At the same time, transient sinus bradycardia and complete AV node block were observed. Symptoms usually faded away within 1 min as normal sinus rhythm and AV node conduction restored. With the use of ventricular pacing backup and low flow oxygen supply, all the patients endured the above symptoms safely. There were no occurrences of bronchoconstriction, oxygen saturation decrease, severe hypotension, and/or malignant arrhythmias.

### Severe Complications of Ablation

One subject in the ATP group with two concealed APs (one at the left anterior free wall, the other at the left posterior septum) suffered from second degree A-V block with 2:1 A-V conduction at the end of ablation, likely due to edema caused by prolonged ablation in the posterior septal area which extended to the atrioventricular node. After 2 days of monitoring and intravenous dexamethasone infusion of 10 mg per day, he made a complete recovery from an A-V block.

One subject in the non-ATP group experienced pericardial tamponade during ablation of an AP at the right free wall of the tricuspid annulus after a steam pop. Pericardial drainage was performed, and the AP was ablated successfully. One subject in the non-ATP group was diagnosed with a hemothorax after ablation with 330 ml of bloody fluid drawn out. Another subject in the non-ATP group was also diagnosed with a hemothorax and recovered with conservative care. Their hemothorax was caused by a sub-clavicular vein puncture for the insertion of a coronary sinus electrode which injured the pleura.

Other patients survived their procedures safely.

### Follow-Up Outcomes

**Figure 5:**
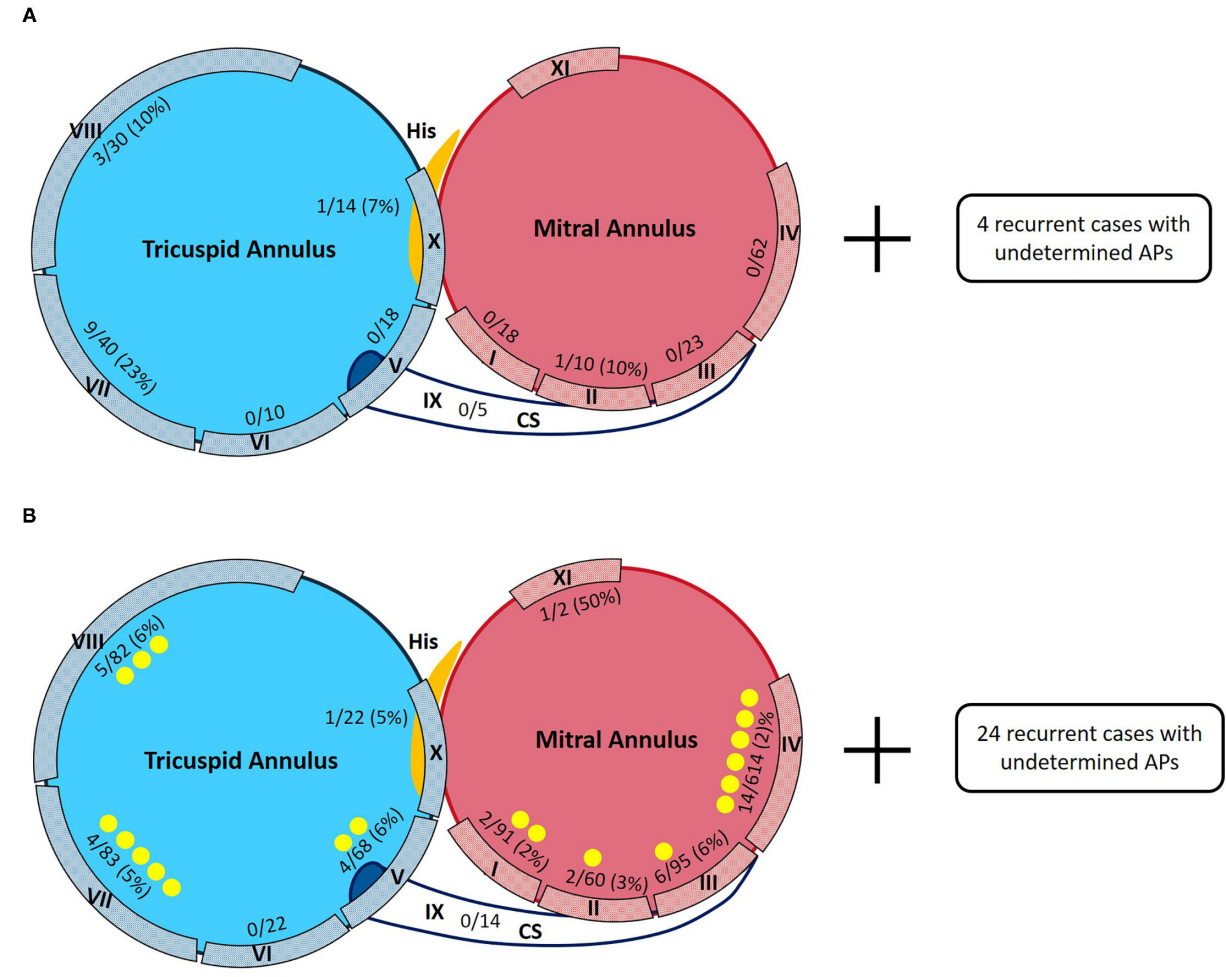
Sketch maps of APs detected in the ATP group and the non-ATP group. Denominators are numbers of APs detected during the first procedures. Numerators are numbers of recurred APs confirmed by redo procedures. Yellow points are for the newly discovered APs during redo procedures. **(A)** Distributions of APs in the ATP group in first and redo procedures. Four recurrent cases with undetermined AP locations were excluded from further analysis. **(B)** Distributions of APs in the non-ATP group in first and redo procedures. Twenty-four recurrent cases with undetermined AP locations were excluded from further analysis.

Among the ATP group, 18 patients (18/214 = 8.4%) had recurrence. There was no significant difference in the recurrence rate between the two groups (*p* = 0.399). Fourteen recurrent patients (14/18 = 77.8%) accepted redo procedures and all were confirmed to have the same APs as their first procedures. Two (2/14 = 14.3%) of them were discovered to have multiple APs, while redo procedures demonstrated that all of them had only 1 recurred AP, 14 in total. The locations and percentages of recurrent APs are listed in Figure 2A and are depicted in Figure 5A. The remaining four recurred cases did not accept redo procedures; however, their surface ECGs showed the same morphology of delta waves as previously seen which suggested that the recurrent APs were likely the same as before. Since the AP locations of these four patients were not confirmed by redo procedures, they were not included in further statistical analysis. On the other hand, 7 out of the 196 patients who did not suffer from recurrence had multiple APs (3.6%).

Among the non-ATP group, 74 patients (74/1,089 = 6.8%) had recurrence. Fifty recurrent patients (50/74 = 67.6%) accepted redo ablations. These patients were discovered to have had a total of 59 APs by redo procedures, including 39 “old” ones and 20 newly detected ones. Twenty (20/50 = 40%) of them were proved to have multiple APs, summed up by their first and redo procedures. Their redo procedures demonstrated one to three recurrent APs for everyone. The locations and percentages of the 59 recurrent APs are listed in Figure 2B and depicted in Figure 5B. The remaining 24 recurrent patients did not accept redo procedures to prove their accurate AP locations; however, the above 9 patients' surface ECGs during follow-up showed the same morphology of delta waves as before, which suggested that the recurrent APs were likely the old ones. These unconfirmed APs were also excluded from further statistical analysis. On the other hand, 33 out of the 1,015 patients who did not suffer from recurrence had multiple APs (3.3%).

New AP locations of recurrent cases confirmed by redo procedures between the two groups were significantly different (*p* = 0.008, by Fisher's exact test, Supplementary Table 3). Existences of multiple APs in the two groups were not significantly different (*p* = 0.415; Supplementary Table 4). Existences of multiple APs in recurred cases were significantly higher than that in non-recurred ones in the non-ATP group (*p* < 0.001; Table 2).

**Table 2:** Differences in existence of multiple APs between recurred and non-recurred cases in both groups.

**Grouping**	**Status**	**Multiple APs (%)**	**Single APs (*p*%)**	**Patients**	***P*-value**
ATP group	Non-recurred	7 (3.6)	189 (96.4)	196	0.114^a^
	Recurred + redo	2 (14.3)	12 (85.7)	14	
	Recurred + non-redo	–	–	4	
Non-ATP group	Non-recurred	33 (3.3)	982 (96.7)	1,015	<0.001^b^
	Recurred + redo	20 (40)	30 (60)	50	
	Recurred + non-redo	–	–	24	

a *Pearson Chi-square value 3.657*.

b *Pearson Chi-square value 136*.

We listed some factors that were potentially related to long-term AP recurrences in both groups including age, gender, body weight, body mass index, hypertension, diabetes, renal insufficiency, hyperurecemia/gout, and baseline structural heart diseases. We analyzed their relations to AP recurrence in both groups, respectively, only to find that male gender and larger body weight were significantly related to higher AP recurrence in the non-ATP group, while none of the above factors were related to recurrence in the ATP group. The results are shown in Table 3.

**Table 3:** Factors potentially related to long-term outcomes of both groups.

**Factors**	**ATP group (*****N*** **= 214)**			**Non-ATP group (*****N*** **= 1,089)**		
	**Recurred(*n* = 18)**	**Non-recurred(*n* = 196)**	***P*-value**	**Recurred(*n* = 74)**	**Non-recurred (*n* = 1,015)**	***P*-value**
Age, years	33.8 ± 15.2	37.2 ± 14.2	0.338	41.1 ± 15.4	41.5 ± 15.0	0.825
Gender, %	M: 11 (61) W: 7 (39)	M: 119 (61) W: 77 (39)	0.963^a^	M: 37 (50) W: 37 (50)	M: 612 (60) W: 403 (40)	0.014^b^
Body weight (kg)	56.6 ± 12.3	61.1 ± 11.1	0.118	57.7 ± 9.8	62.5 ± 11.6	0.009
Body mass index (kg/m^2^)	20.9 ± 2.7	22.5 ± 3.4	0.227	22.2 ± 3.3	23.0 ± 5.1	0.284
Hypertension	0	17 (8.7)	0.371^d^	7 (9.5)	84 (8.3)	0.749^c^
Diabetes	0	5 (2.6)	1^d^	3 (4.1)	31 (3.1)	0.499^d^
Renal insufficiency	0	1 (0.5)	1^d^	1 (1.4)	2 (0.2)	0.19^d^
Hyperglycemia or gout	1 (5.6)	6 (3)	0.464^d^	4 (5.4)	25 (2.5)	0.101^d^
Baseline structural heart diseases	2 (11.1)	5 (2.6)	0.109^d^	1 (1.4)	57 (5.6)	0.175^d^

a *Pearson Chi-square value 0.002*.

b *Pearson Chi-Square value 6.072*.

c *Pearson Chi-Square value 0.102*.

d *Analyzed by Fisher's Exact Test*.

*Renal dysfunction was defined as eGFR <60 ml/min. Since the enrollment of both groups was not decided randomly, no comparison was made between groups*.

## Discussion

The major findings of the present study are as follows:

1. The existence of multiple APs increases recurrent risk if ATP was not used for confirmation of ablation endpoints.
2. Sufficient ATP not only unmasks dormant AP conduction after initial ablation, but also reveals weak APs that are less conductive than the AV node under PES.
3. ATP can evoke atrial and ventricular arrhythmias.
4. ATP bolus injection is safe with ventricular back-up pacing.

It has long been proven that existence of multiple APs is a risk factor for recurrence after ablation (7). This study also attained similar results. While our main discovery was that sufficient ATP administration for endpoint confirmation of AP ablation probably improved long-term outcomes.

ATP can be rapidly degraded into adenosine diphosphate (ADP), with ADP degraded into adenosine monophosphate (AMP) and AMP dephosphorylated into adenosine, it is conceivable that some of the ATP effects on the AV node are mediated by adenosine. ATP has a similar function to adenosine which can block AV nodal conduction transiently and reversibly but seldom blocks AP conduction (except for those rare AV node-dependent APs, such as nodo-ventricular and nodo-fascicular APs). The half-life of ATP is about 30 s. The effect of both adenosine and ATP on the AV node resembles the action of slow channel blocking agents, such as verapamil and manganese chloride (8). It was suggested that the AV block caused by adenosine is associated with suppression of isoproterenol-enhanced I_f_ and I_ca_ (9). The rapid onset and transient duration of the adenosine- and ATP-induced AV block are also similar to the effects of acetylcholine on the AV node (10). Adenosine and ATP have long been utilized in emergency rooms to terminate narrow-QRS tachycardias (11, 12). Since ATP solely blocks AV nodal conduction, it can be useful for the detection of extra-nodal pathways. It is additionally useful in EP labs for the differential diagnosis between supraventricular tachycardia and ventricular tachycardia and for testing of ablation endpoints of APs.

Besides its blocking function, ATP has also been reported to provoke dormant electrical conduction. It can provoke reconnection between the left atrium and the pulmonary veins after initially successful pulmonary vein isolation (13). The mechanism of this phenomenon is thought to be secondary to membrane hyperpolarization of partially depolarized cardiac tissue caused by ablation impairment (14). It can also induce atrial tachycardias (15) and premature ventricular ectopies (16, 17) by its potential to activate muscular sleeve fibers. The above phenomena were also observed in our series of patients. We observed episodes of AF after ATP injection in several patients. The detailed mechanism of ATP inducing arrhythmias is not clear, but some believe that it mainly depends on the ATP-sensitive K^+^ channels. Li and colleagues reported that adenosine-induced AF is driven by localized reentry in RA areas with highest expression of adenosine A1 receptors and its downstream G protein-coupled inwardly rectifying potassium (GIRK) channels (I_K,Ado_) (18). Such research has suggested that there exists some molecular basis for ATP and adenosine to induce arrhythmias. In our opinion, ATP is helpful in the induction of certain arrhythmias which are due to activation of localized muscular sleeves. There were also reports on adenosine unmasking recurrent dormant AP conduction right after catheter ablation (5, 6). Dormant AP conduction was reported to be associated with higher rates of recurrent AP conduction requiring repeat ablation and possibly via AP membrane potential hyperpolarization. Alvarez prospectively applied 108 ATP tests on Wolff-Parkinson-White patients undergoing ablation procedures. After successful ablation confirmed by PES, ATP was injected. The diagnostic accuracy of the ATP test was 95%, sensitivity 69%, specificity 100%, and positive and negative predictive values 100 and 95%, respectively. They suggested that ATP administration has a high predictive value for AP early recurrence (19). However, none of these studies had control groups and reported the results of long-term follow-up.

Our study also observed the same phenomena as above. Most APs detected during redo procedures in both groups were initial ones. There were 7.4% of cases in the ATP group that demonstrated having dormant APs right after injection of ATP. However, we still observed something new. All the APs of the recurrent cases in the ATP group were initial ones, while only 66% of the APs of recurrent cases in the non-ATP group were initial ones. In addition, ATP also discovered new APs in five cases after initial ablations.

All these patients continued with ablation until they passed both tests as a reinforced endpoint. This phenomenon suggested that injured APs were likely to “hide” themselves during routine PES, especially when there was more predominant conduction through the AV node. Some inactive APs may also remain undiscovered during PES if AV nodal conduction was superior until the AV node was blocked by ATP. This phenomenon may be due to three factors. One is that the prolonged refractory period of injured APs prevents detection by PES. Another is the activating effect of ATP on AP bundles. The third is the prolonged waiting time due to PES plus ATP testing. All of the above suggested that passing PES plus ATP testing as a combined endpoint of AP ablation was helpful to successfully eliminate APs. Although a full-dosage ATP bolus aiming to result in complete AV block can cause obvious transient side effects, it is nevertheless safe under the protection of ventricular pacing and by avoiding the contraindications to this agent, including asthma, glaucoma, severe urinary obstruction, etc.

Recurrence rates of AP conduction after successful ablation were 5–8% according to past studies (20–22). In our study, the recurrence rate was 8.4% in the ATP group and 6.8% in the non-ATP group, like others. The fact was that the recurrence rate was higher in the ATP group than in the non-ATP group in our study, although not statistically significant, suggesting that the combined procedural endpoint did not seemingly benefit long-term prognosis. We consider this finding to be mainly caused by selection bias of the operating physicians who were prone to use ATP in more challenging cases subjectively, such as those APs on the free wall of the tricuspid annulus, APs within CS, APs on AMC, etc. Since ATP testing helps us to observe many extra electrophysiologic phenomena during ATP testing, especially evidence of new APs which had not been discovered by routine PES and reappearance of ablated APs, we can still conclude that ATP provides additional value over PES alone in confirmation of an ablation endpoint for APs. Moreover, the recurrent APs within the ATP group during long-term follow-up were the same ones, while the recurrent APs within the non-ATP group during follow-up were prone to be newly detected ones. This difference between the two groups was statistically significant. This also suggested that ATP testing may help discover all APs present at an initial ablation procedure, potentially resulting in better long-term outcomes.

A past study administered a small bolus of ATP to patients with APs. They found that failure of an intravenous bolus injection of 8 mg of ATP to produce V-A conduction block identified the presence of an AP with a sensitivity of 84%, specificity of 71%, and predictive value of 72%. The effects of ATP on the AV node were concordant with the effects of a combination of verapamil and propranolol in 91% of patients, suggesting that this dose of ATP was an equipotent AV nodal blocker with a short duration of action (23). We considered an 8-mg ATP bolus via peripheral veins inadequate to block the AV node completely. It is more likely to terminate supraventricular tachycardia by alternating AV nodal refractory periods than causing complete AV nodal block which is required for AP detection in the EP lab. Under the protection of ventricular pacing, a larger bolus of ATP between 30 and 40 mg is safe even when complete AV block occurs with a resultant slow ventricular escape rhythm. ATP's very short half-life also safeguards its utilization.

We also tried to find out the traditional factors that were potentially related to AP recurrence. We only found that male gender and larger body weight were significantly related to higher AP recurrence in the non-ATP group, while none of the above factors were related to recurrence in the ATP group. But we do not consider this result as meaningful.

## Limitations

The main limitation of this study is its retrospective design and personal selection bias based on selective ATP application by different operators. Based on the positive findings during ATP testing in addition to PES, this study is still representative in a way. Further prospective studies are necessary to obtain a more conclusive determination of the benefits of such testing.

## Conclusions

The existence of multiple APs was more common in recurrent cases if ATP was not used for confirmation of ablation endpoints. ATP probably adds additional value over PES alone by detecting weak AP conductions. ATP can evoke atrial and ventricular arrhythmias. With the use of temporary ventricular back-up pacing, higher dosage ATP testing is safe in patients without contraindications to this agent.

## Data Availability Statement

The raw data supporting the conclusions of this article will be made available by the authors, without undue reservation.

## Ethics Statement

The studies involving human participants were reviewed and approved by the Research Ethics Committee, Guangdong Provincial People's Hospital, Guangdong Academy of Medical Sciences. Written informed consent to participate in this study was provided by the participants' legal guardian/next of kin.

## Author Contributions

WW and XF collected the data and wrote the manuscript. MS and XW polished the language. XZ, HL, HD, and ZL did the procedures. YL did statistics. YX and SW funded the research. All authors contributed to the article and approved the submitted version.

## Funding

This study was funded by grants from the National Natural Science Foundation of China (81900301), the National Key Research and Development Program of China (2018YFC1312502), the Science and Technology Planning Project of Guangzhou (201904010451), and the Special Project for Research and Development in Key Areas of Guangdong Province (2019B020230004).

## Conflict of Interest

The authors declare that the research was conducted in the absence of any commercial or financial relationships that could be construed as a potential conflict of interest.

## Publisher's Note

All claims expressed in this article are solely those of the authors and do not necessarily represent those of their affiliated organizations, or those of the publisher, the editors and the reviewers. Any product that may be evaluated in this article, or claim that may be made by its manufacturer, is not guaranteed or endorsed by the publisher.

## Supplementary Material

Supplementary Figure 1 Preset location distributions of accessory pathways. I: left posterior septum, II: left posterior free wall, III: left lateral free wall, IV: left anterior free wall, V: right posterior septum (coronary sinus orifice), VI: 6 o'clock in tricuspid annulus, VII: 6–9 o'clock in tricuspid annulus, VIII: 9–12 o'clock in tricuspid annulus, IX: proximal coronary sinus including middle cardiac vein (epicardial), X: para-Hisian accessory pathways (target with H potential), AMC: aortomitral continuity.

Supplementary Table 1 Distributions of all detected APs in both groups during the index procedure.

Supplementary Table 2 Results of ATP testing at the first procedures.

Supplementary Table 3 Difference in recurrent AP locations confirmed by redo procedures between the two groups.

Supplementary Table 4 Differences in existence of multiple AP cases in both groups.

Supplementary Table 5 Differences in existence of multiple APs between recurred and non-recurred cases in both groups.
