# Supplementary figures and images for: Administration of Adenosine Triphosphate Provides Additional Value Over Programmed Electrophysiologic Study in Confirmation of Successful Ablation of Atrioventricular Accessory Pathways
Source: Front Cardiovasc Med. 2021 Nov 16;8:716400. doi: 10.3389/fcvm.2021.716400 (PMC8635057; doi:10.3389/fcvm.2021.716400)

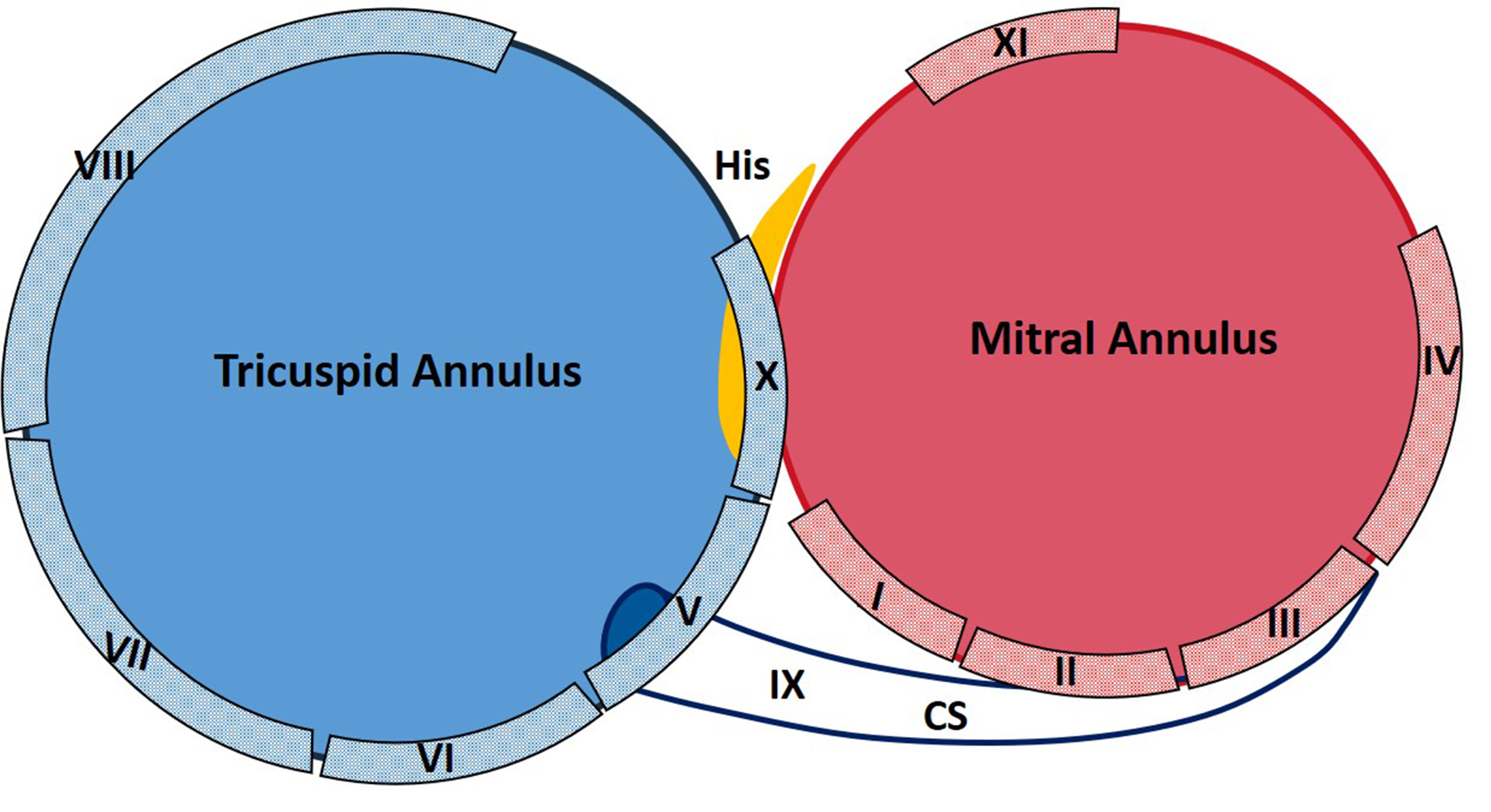

Supplement: Supplementary Figure 1 — Preset location distributions of accessory pathways. I: left posterior septum, II: left posterior free wall, III: left lateral free wall, IV: left anterior free wall, V: right posterior septum (coronary sinus orifice), VI: 6 o'clock in tricuspid annulus, VII: 6–9 o'clock in tricuspid annulus, VIII: 9–12 o'clock in tricuspid annulus, IX: proximal coronary sinus including middle cardiac vein (epicardial), X: para-Hisian accessory pathways (target with H potential), AMC: aortomitral continuity. [file Image_1.JPEG]
