# Supplementary material for: Administration of Adenosine Triphosphate Provides Additional Value Over Programmed Electrophysiologic Study in Confirmation of Successful Ablation of Atrioventricular Accessory Pathways
Source: Front Cardiovasc Med. 2021 Nov 16;8:716400. doi: 10.3389/fcvm.2021.716400 (PMC8635057; doi:10.3389/fcvm.2021.716400)
Supplement: Supplementary Table 1 — Distributions of all detected APs in both groups during the index procedure. [file Data_Sheet_1.docx]

**Supplemental Table 1 Distributions of all detected APs in both groups during the index procedure**

| **AP locations** | | **I** | **II** | **III** | **IV** | **V** | **VI** | **VII** | **VIII** | **IX** | **X** | **AMC** | **NV** | **Total AP number** |
| --- | --- | --- | --- | --- | --- | --- | --- | --- | --- | --- | --- | --- | --- | --- |
| **ATP** | **No.** | 18 | 10 | 23 | 62 | 18 | 10 | 40 | 30 | 5 | 14 | 2 | 1 | 232 |
|  | **%** | 7.7% | 4.3% | 9.9% | 26.7% | 7.7% | 4.3% | 17.2% | 12.9% | 2.1% | 6.0% | 0.9% | 0.4% | 100.0% |
| **Non-ATP** | **No.** | 91 | 60 | 95 | 614 | 68 | 22 | 83 | 82 | 14 | 22 | 2 | 0 | 1153 |
|  | **%** | 7.9% | 5.2% | 8.2% | 53.3% | 5.9% | 1.9% | 7.2% | 7.1% | 1.2% | 1.9% | 0.2% | 0 | 100.0% |
| **Sum** | | 109 | 70 | 118 | 676 | 86 | 32 | 123 | 112 | 19 | 36 | 4 | 1 | 1385 |

The distributions of all the AP locations within the two groups were significantly different (Pearson Chi-square value= 81.785, p<0.001).

AMC: [aortomitral continuit](https://www.ncbi.nlm.nih.gov/pubmed/21979993)y, NV: nodo-ventricular fiber

**Supplemental Table 2 Results of ATP testing at the first procedures**

| Phenomena | Cases (%)  (N=215) |
| --- | --- |
| Re-appearance of initial APs | 16 (7.4) |
| Traces of other APs | 5 (2.3) |
| Paroxysmal atrial fibrillation | 5 (2.3) |
| Atrial tachycardia/premature atrial contractions | 1 (0.5) |

**Supplemental Table 3 Difference in recurrent AP locations confirmed by redo procedures between the two groups.**

| Grouping | Same APs | Different APs | Total |
| --- | --- | --- | --- |
| ATP group | 14 | 0 | 14 |
| Non-ATP group | 39 | 20 | 59 |
| Total | 53 | 20 | 73 |

*p*=0.008, by Fisher’s exact test.

**Supplemental Table 4 Differences in existence of multiple AP cases in both groups**

| Grouping | Status | Multiple APs (%) | Single APs (p%) | Patients | P value |
| --- | --- | --- | --- | --- | --- |
| ATP group | Proved  Unproved (Recurred+nonredo) | 9 (4.3)  - | 201 (95.7)  - | 210  4 | 0.415 |
| Non-ATP group | Proved  Unproved (Recurred+nonredo) | 53 (5)  - | 1012 (95)  - | 1065  24 |  |

Pearson Chi-square value 0.181.

**Supplemental Table 5 Differences in existence of multiple APs between recurred and non-recurred cases in both groups**

| Grouping | Status | Multiple APs (%) | Single APs (p%) | Patients | P value |
| --- | --- | --- | --- | --- | --- |
| ATP group | Nonrecurred  Recurred+redo  Recurred+nonredo | 7 (3.6)  2 (14.3)  - | 189 (96.4)  12 (85.7)  - | 196  14  4 | 0.114^a^ |
| Non-ATP group | Nonrecurred  Recurred+redo  Recurred+nonredo | 33 (3.3)  20 (40)  - | 982 (96.7)  30 (60)  - | 1015  50  24 | <0.001^b^ |

**a: Pearson Chi-square value 3.657; b: Pearson Chi-square value 136.**
